# Effects of Volume-Price Contracts on Pharmaceutical Prices: A Retrospective Comparative Study of Public Hospitals in Hubei of China

**DOI:** 10.3389/fphar.2021.741671

**Published:** 2021-10-14

**Authors:** Zhuoxian Li, Chaojie Liu, Keyuan Zuo, Junjie Liu, Yuqing Tang

**Affiliations:** ^1^ School of Medicine and Health Management, Tongji Medical College, Huazhong University of Science and Technology, Wuhan, China; ^2^ School of Psychology and Public Health, La Trobe University, Melbourne, VIC, Australia; ^3^ Hubei Public Resource Trading Center, Wuhan, China; ^4^ School of Statistics and Mathematics, Central University of Finance and Economics, Beijing, China

**Keywords:** volume-price contract, collective procurement, competition, pharmaceutical, China, price

## Abstract

**Background:** Pharmaceutical expenditure has been increasing worldwide. Many countries have attempted to contain the increase through collective bargaining, including in China. In 2015, the Chinese government introduced a new policy to empower regional governments to reduce pharmaceutical prices through its existing tendering system which enables a lower price for products with higher procurement volumes. Xiangyang municipality in Hubei province took a lead in piloting this initiative.

**Objectives:** This study aimed to evaluate the effects of the volume-price contract initiative on pharmaceutical price procured by the public hospitals in Xiangyang.

**Methods:** A retrospective comparative design was adopted. The price of cardiovascular medicines (349 products under 164 International Nonproprietary Names) procured by the public hospitals in Xiangyang was compared with those procured in Yichang municipality in Hubei. A total of 15,921 procurement records over the period from January 2017 to December 2018 were examined (Xiangyang started the volume-price contract initiative in January 2018). Generalized linear regression models with a difference-in-differences approach which could reflect the differences between the two cities between January 2018 and December 2018 were established to test the effects of the volume-price contract initiative on pharmaceutical prices.

**Results:** On average, the procurement price for cardiovascular medicines adjusted by defined daily dosage in Xiangyang dropped by 41.51%, compared with a 0.22% decrease in Yichang. The difference-in-differences results showed that the volume-price contract initiative resulted in a 36.24% drop (*p* = 0.006) in the price (30.23% for the original brands, *p* = 0.008), in addition to the therapeutic competition effect (31.61% reduction in the price, *p* = 0.002). The top 100 domestic suppliers were highly responsive to the initiative (82.80% drop in the price, *p* = 0.001).

**Conclusion:** The volume-price contract initiative has the potential to bring down the price of pharmaceutical supplies. Higher responses from the domestic suppliers are evident.

## Introduction

Pharmaceuticals account for a profound share of total health expenditure, ranging from an average of 19.7% in high-income countries to 30.4% in low-income countries (Ye et al., 2011). This proportion was around 14.17% in European countries in 2018 (Eurostat, 2021). A rapid growth in pharmaceutical spending is a worldwide concern. The Intercontinental Medical Statistics (IMS) estimated that pharmaceutical expenditure has been increasing at a speed significantly higher than that of global economic growth. From 2010 to 2015, there was an annual growth of 6.2% in global spending on medicines, rising from $US887 billion to $US1069 billion (IMS, 2015). It is projected to exceed $US1.1 trillion in 2024 (IQVIA, 2020).

Soaring pharmaceutical expenditure imposes a great burden on government budgets, which has triggered a range of policy, regulatory and managerial interventions. Collective purchasing has been used as a tool worldwide to lower the price of pharmaceutical supplies (Dylst et al., 2011; Maniadakis et al., 2018). It forces suppliers to compete for the right to become a dominant supplier in certain markets in line with some strictly predefined criteria (Maniadakis et al., 2018). A purchaser can increase its bargaining power by widening the network of collective purchasing. South Africa, for example, introduced a national tendering system for pharmaceuticals in 1982. Empirical evidence showed that the price of pharmaceutical supplies covered by the tendering system dropped by an average of 40% or more between 2003 and 2016 (Wouters et al., 2019). In Mexico, a commission to purchase antiretrovirals and other medicines achieved a cost saving of $US52.1–121.8 million in its first 4 years since inception in 2008 (Gomez-Dantes et al., 2012; Adesina et al., 2013). Cost savings were also found through collective purchasing at the subnational levels, such as the Intermunicipal Health Consortium in Brazil (de Amaral and Blatt, 2011) and the hospital networks in Serbia (Milovanovic et al., 2004) and Brazil (Sigulem and Zucchi, 2009). Collective tendering in European countries has been proved to enhance competition, resulting in reduced prices in pharmaceutical supplies (Dylst et al., 2011; van Woerkom et al., 2012; Vogler et al., 2017; Jensen et al., 2020). In China, rising pharmaceutical expenditure has attracted a great deal of policy attention over the past decade. From 2010 to 2017, pharmaceutical expenses as a proportion of health expenditure in China declined from 41.6% to 34.4% (National Health Commission, 2019). However, it remained at a high level in comparison with OECD (Organization for Economic Cooperation and Development) countries (18.2% in 2010 and 16.1% in 2017) (OECD, 2020). The actual pharmaceutical spending over the same period increased from 883.59 billion yuan ($US129.45 billion) to 1820.30 billion yuan ($US266.67 billion) (National Health Commission, 2019).

Traditionally, pharmaceutical policy debates in China were centered around caps in pharmaceutical prices and mark-up margins allowed for health providers (Hasan et al., 2019; Liu et al., 2019). The Chinese government categorized pharmaceutical products into two groups. Group A are mainly prescription medicines while Group B are mainly over-the-counter medicines (National Development and Reform Commission, 2005). The National Development and Reform Commission (NDRC) imposed a price cap for Group A medicines based on the declared costs from the manufacturers. The provincial governments imposed a price cap for Group B medicines under the guiding prices developed by the NDRC (National Development and Reform Commission, 2005; Hasan et al., 2019). Between 1997 and 2013, over 30 mandatory regulations mostly related to price caps on medicines were announced. Empirical studies showed that these policies were not as effective as anticipated. Pharmaceutical manufacturers could easily evade price caps by registering their products as innovative new drugs through some minor modifications such as dosage forms (Wu et al., 2015; Hu and Mossialos, 2016). This is not unique to China (Vernaz et al., 2013). Meanwhile, the 15% mark-up rule for health institutions provided perverse incentives for medical doctors to prescribe more expensive medicines such as injections and traditional Chinese medicines (Meng et al., 2005; Zeng et al., 2014; Zeng et al., 2015; Hu and Mossialos, 2016). Eventually, the NDRC abolished the price cap policy in 2015 (Hu and Mossialos, 2016). Markups for health institutions on sales of medicines have been officially removed since 2017 throughout the country (Tang et al., 2018). As a result, there are high expectations that centralized tendering and collective purchasing which is gradually developing towards volume-price contract initiative will play a significant role in curtailing the pricing inflation of pharmaceutical products (Hu and Mossialos, 2016).

The exploration of centralized tendering and collective procurement of medicines in China dates back to the 1990s. But it was not until 2009 that it became a nationwide province-based governmental practice. The centralized procurement arrangements started with essential medicines for primary care and were gradually extended to pharmaceutical procurements for public hospitals. Each provincial government has its own online platform, supporting tendering, contracting, purchasing, and distribution of pharmaceutical products (Cai, 2017). Since 2010, each tender has been required to submit two separate bidding documents (“two-envelope”) demonstrating its bidding price and quality of products and services, respectively (Hu and Mossialos, 2016). The winners were supposed to go with the suppliers with the highest composite score of the two envelopes (although usually the lowest price won) (Hu et al., 2019). In some provinces, only one supplier would be contracted to supply certain medicines, while in other provinces, two or more suppliers could be contracted (Hu and Mossialos, 2016). It was estimated that the price of essential medicines for primary care decreased by an average of 25% and even over 50% in some provinces between 2009 and 2010 (Hu, 2013).

Despite the overall drop in prices, the procurement system was criticized for its lack of capacity to link price with volumes of purchased medicines (Fu et al., 2015). The tendering systems overseen by the provincial governments were only responsible for identifying contracted suppliers and settling the prices of pharmaceutical products. No procurement volumes were announced specifically in the tendering. It was up to each individual health institution to make monthly purchase orders and to settle on delivered prices through a “second bargaining” with the suppliers. In addition, there was a lack of supervision over the procurement contracts signed between the health institutions and the suppliers. As a result, pharmaceutical suppliers were placed in a financial dilemma since they could not properly establish an offer based on an accurate estimation of the market share (Tang et al., 2017). Some awarded suppliers would simply not deliver purchase orders if their bidding price was deemed too low to cover the costs. This was particularly common for the lowest-priced generic medicines (Dylst et al., 2013; Fang et al., 2013). Meanwhile, suppliers were likely to manage the risk of market uncertainty through inflating prices, especially for high-priced products (Jiang et al., 2014).

In 2015, the central government issued two policy documents, instructing provincial governments to rationalize procurement prices by attaching procurement volumes to prices in procurement contracts (General Office of the State Council, 2015; National Health and Family Planning Commission, 2015). In practice, it is up to each provincial government to decide the scope for the volume-price contract initiative. Provincial governments continue to set up the highest prices allowed for the included pharmaceutical products. However, health institutions are grouped (selected or all-inclusive at a municipal level or across several municipalities) to bargain for further lower prices for a collective volume of purchase orders. The procurement procedures have to be carried out on the provincial online procurement platform (Li et al., 2018).

Hubei started to pilot the volume-price contract initiative in 2016 in three municipalities: Wuhan, Xiangyang and Ezhou. The municipal governments were authorized to develop their own pharmaceutical catalogues covered in the initiative. However, the procurement volume of each pharmaceutical product had to be justified with reference to its consumption in the previous year (Government of Hubei Province, 2016). For each pharmaceutical product defined by molecule structure, formulation and strength, no more than two suppliers could be awarded. The government used this strategy to make the tendering more attractive (less suppliers and less competition) to those who were willing to reduce price (Government of Hubei Province, 2016). In 2017, the Hubei government issued policy instructions on the volume-based procurement procedure as a condition to sign volume-price contracts (Health Commission of Hubei Province, 2017). This study aimed to evaluate the price effect of the volume-price contract initiative on pharmaceutical supplies to public hospitals in Xiangyang municipality of Hubei province. The findings would be helpful for both researchers and policy makers since such empirical evidence is still lacking as far as we know.

## Methods

### Study Setting

A retrospective comparative study was conducted in Hubei province. The volume-price contract initiative implemented in Xiangyang municipality was evaluated, with Yichang municipality serving as the control.

Hubei covers an area of 185,900 km^2^ and has about 59.02 million residents. Its per capita annual disposable income reached 31,889 yuan ($US4672) for urban and 13,812 yuan ($US 2023) for rural residents in 2017, 87.62% and 102.83% of the national average, respectively (Bureau of Statistics of Hubei Province, 2018a). About 5.89% of GDP (gross domestic product) was spent on health (192.472 billion yuan, or $US28.200 billion) in 2016. There were approximately 2.50 registered physicians, 3.10 nurses, and 6.37 hospital beds per 1,000 people across 36,357 health care institutions in the province in 2017 (National Health Commission of China, 2018).

Xiangyang occupies a comparable land size (19,728 km^2^) as Yichang (21,084 km^2^), but with more dense dwelling. The GDP in Xiangyang ranked second among all municipalities in Hubei in 2017, while Yichang ranked third (Bureau of Statistics of Hubei Province, 2018a). However, the disposable income per capita of all residents in Xiangyang ($US3520) was slightly lower than that of Yichang ($US3543) in 2017. Xiangyang had more health resources and spent more on health compared with Yichang (Table 1) (Bureau of Statistics of Hubei Province, 2018c; b).

**TABLE 1:** Socioeconomic characteristics of the two study municipalities in 2017.

Socioeconomic indicator	Xiangyang (intervention)	Yichang (control)
Gross Domestic Product (GDP, 100 million CNY)	4,064.90	3,857.17
Per capita GDP (CNY)	71,990	93,331
Population size (Million)	5.654	4.136
Per capita disposable income (CNY)	24,030	24,182
Number of health institutions	3,731	3,013
Number of hospital beds	36,507	28,180
Number of licensed (assistant) doctors	13,934	10,919
Number of skilled health workers	39,591	38,275
TOPSIS score	0.378043889	0.341987109

Note: *CNY -* Chinese Yuan, *TOPSIS -* technique for order performance by similarity to ideal solution.

Yichang was selected as a control group through a comprehensive assessment of all 17 municipalities in Hubei using an unweighted TOPSIS (technique for order performance by similarity to ideal solution) method (Tang et al., 2016) (Table 1 in the Supplementary Material). Yichang had the closest match (TOPSIS score) with Xiangyang considering eight matching variables: GDP, per capita GDP, population size, per capita disposable income, number of health institutions, number of hospital beds, number of licensed (assistant) doctors, and number of skilled health workers (Table 1). These indicators can reflect the level of economic and health system development (Bureau of Statistics of Hubei Province, 2018a; c; b).

Supplementary material Table S1 Results of (unweighted) TOPSIS ranking.

### Study Design and Data Sources

A retrospective comparative with a difference-in-differences approach was adopted. A total of 15,921 procurement records for cardiovascular medicines over the period from January 2017 to December 2018 were examined. Cardiovascular medicines were chosen in this study since it is a key area with the growing prevalence of cardiovascular diseases and multiple medicines relevant to the treatment of cardiovascular diseases (Mirsafaei et al., 2020; Wei et al., 2020). In addition, they accounted for a large proportion of procurements for specialized medicines, and the volume-price contract initiative for cardiovascular medicines was mature and had a clear cut implementation in January 2018 (Centralized Pharmaceuticals Tendering and Procurement Center of Xiangyang, 2017). In contrast, Yichang, the control group, had not introduced the volume-price contract system over the entire study period. This enabled us to compare the procurement records in the two municipalities before and after the new initiative.

Data came from the Hubei Medical Procurement Administrative Procurement System (HMPAPS). Eligible records were identified using the anatomical therapeutic chemical (ATC) classification and coding system. We restricted the study sample to cardiovascular medicines with an ATC code C. A total of 15,921 procurement records over the study period for cardiovascular medicines were extracted, covering 164 International Nonproprietary Names (INN) and 349 products. These medicines were procured for the 35 public hospitals in the two municipalities: 21 in Xiangyang and 14 in Yichang. Medication needs depended on the local population and their health profiles. The local governments and medical institutions were delegated with the power to select the medicines in line with their local clinical needs. Data items extracted included: procurement serial number, hospital name, time of procurement, INN, formulations, strength, package size, procurement price per package (CNY, Chinese Yuan), procurement volume (packages), procurement cost (CNY), and suppliers of different medicines. We further classified the pharmaceutical products into subgroups according to their brand (original brand and generic) and administration routes (injectable and oral). The suppliers were categorized by ownership (domestic, joint venture, foreign-owned) and ranking of financial outputs (Southern Medicine Economic Research Institute of National Medical Products Administration, 2018; 2019).

### Intervention Measures

The Xiangyang municipality (intervention group) introduced the volume-price contract initiative through a staggered approach, starting with several proton pump inhibitors (digestive medicines) at the beginning of 2017, followed by antimicrobial medicines and patent Chinese medicines in August 2017. Lessons learnt from the two stages of implementation were fed into the final stage of implementation in January 2018, targeting a broad range of specialized medicines including cardiovascular medicines. Procurement of the relevant medicines from all of the 21 public hospitals in Xiangyang were pooled for a better bargaining price based on the large pooled volume. Tendering was organized with a promised volume for each procured medicine calculated based on the clinical needs for six or more months (Health Commission of Hubei Province, 2017). The price of each procured medicine (with specified INN, dosage form, strength, pack size) under the volume-price contract was fixed for all of the covered local health institutions, which lasted for 1 year. In the volume-price contract system, the tendering and procurement procedures were integrated.

Over the study period, the Yichang municipality (control group) maintained its existing procurement system similar to that of Xiangyang prior to the introduction of the volume-price contract system. The provincial government organized the tendering and determined the awarded suppliers without guaranteed procurement volumes. Each individual hospital then conducted its own “second bargaining” for the price of purchase orders (Health and Family Planning Commission of Hubei Province, 2014). Under such a system, the governments were only responsible for identifying suppliers through tendering, leaving the actual procurements in the hands of individual health institutions.

### Statistical Analysis

Price change was the primary interest of this study, which was the major policy goal of the volume-price contract initiative. The unit price of each procured medicine was calculated based on its defined daily dosage (DDD) defined by World Health Organization in 2018 (WHO Collaborating Centre for Drug Statistics Methodology, 2018) in absolute monetary terms (CNY).

Generalized linear regression models with a difference-in-differences approach were established to determine price (non-normal distribution) changes associated with the volume-price contract initiative:

$$Y_{ijt}=\alpha_{0}+\beta \times Policy_{ijt}+\gamma \times X_{ijt}+\alpha_{j}+\delta_{t}+\varepsilon_{ijt}$$

where *Y* indicates the unit price of procured medicines, *i* indicates a specific cardiovascular product, *j* represents the public hospital, and *t* indicates the month (24-month periods). $\alpha_{j}$ and $\delta_{t}$ are fixed effects of hospitals and months, respectively. $\varepsilon_{ijt}$ refers to the random error term. The difference-in-differences effect of the volume-price contract initiative was measured by the regression coefficient *β* for $Policy_{ijt}$ , an interaction term between the study group (1 = Xiangyang, 0 = Yichang) and the time (1 = 2018, 0 = 2017).

Several indicators were calculated to measure competition ( $X_{ijt}$ ), a dominant force for price setting in a market system. These included generic competition (the number of different products that had the same molecule defined by the ATC fifth-level code) and therapeutic competition (the number of different products that were in the same therapeutic subgroup defined by the ATC fourth-level code) (Zhao and Wu, 2017). Given the possibility of the nonlinear effects of competition (Spinks et al., 2013; Liu et al., 2017), a squared term of each competition indicator was added to the regression models.

We established modelling for the entire sample, as well as modelling for the subsamples categorized by ownership of suppliers and characteristics of medicines in line with the Hedonic model (Rosen, 1974). The estimation of standard errors in the modeling for the sample were clustered at the level of cardiovascular products (procurement serial numbers).

Modified Park tests and Box-Cox tests were used to estimate family distribution and link function of the outcome indicator (unit price), respectively. Log link and Gamma distribution were applied in the modelling for the entire sample and most subsamples, except for the subsample containing foreign-owned suppliers only and the subsample containing original brands only, for which log link and inverse Gaussian distribution were applied. In addition, square root link and Gamma distribution were applied in the modelling for the subsample containing oral medicines only (Table 2).

**TABLE 2:** Estimation of link function (Park tests) and family distribution (Box–Cox tests) of unit price of pharmaceutical products.

Study sample	Modified park test for family distribution		Box-cox test for link function	
**Total**	1.77	Gamma	−0.13	Log
**Subsample by ownership**				
Domestic suppliers outside of top 100	1.98	Gamma	−0.07	Log
Top 100 domestic suppliers	2.02	Gamma	−0.20	Log
Suppliers with joint venture	2.09	Gamma	−0.24	Log
Foreign-owned suppliers	3.53	Inverse gaussian	−0.33	Log
**Subsample by medicines**				
Original brand	3.53	Inverse gaussian	−0.25	Log
Generic	1.84	Gamma	−0.10	Log
**Subsample by administration routes**				
Injectable	2.00	Gamma	0.03	Log
Oral	1.72	Gamma	0.42	Square root

Note: Coefficients excerpted from modified Park tests: 0 = Gaussian distribution (variance unrelated to the mean); 1 = Poisson distribution (variance equal to the mean); 2 = Gamma distribution (variance exceeding the mean); 3 = Inverse Gaussian distribution (or Wald distribution). Coefficients excerpted from Box–Cox tests: 0 = log link; 0.5 = square root link; 1 = identity link.

## Results

### Supply of Cardiovascular Medicines

Table 3 shows changes to the procured cardiovascular medicines before and after the new initiative in the intervention group (Xiangyang) and the control group (Yichang). There were no obvious changes in the number of INNs and type of products in the control group. But the type of products dropped by 38.86%, from 229 before the initiative to 140 after the initiative; and the number of INNs dropped by 26.98%, from 126 down to 92 in the intervention group. Meanwhile, the monthly average number of both generic (changing from 1.86 to 1.09) and therapeutic (changing from 6.63 to 3.84) competitors per product also declined in the intervention group, compared with an increase in the control group (changing from 2.29 to 2.41 for generic competitors and from 7.99 to 8.71 for therapeutic competitors). Overall, the share of different medicines (generic vs original brands; oral vs injectable) and suppliers (by ownership and financial outputs) remained unchanged: the vast majority of the market was occupied by generic and oral medicines and domestic suppliers.

**TABLE 3:** Supply of cardiovascular medicines in the participating municipalities over the study period (2017–2018).

Characteristics of supply	Xiangyang (intervention)		Yichang (control)	
	2017	2018	2017	2018
Number of INNs	126	92	132	130
Type of procured products	229	140	233	236
Generic competitors per product per month (Mean ± SD)	1.86 (±1.56)	1.09 (±1.19)	2.29 (±2.11)	2.41 (±2.01)
Therapeutic competitors per product per month (Mean ± SD)	6.63 (±5.19)	3.84 (±3.65)	7.99 (±5.64)	8.71 (±6.14)
**Number (%) of products supplied by:**				
Domestic suppliers outside of top 100	141 (61.57)	78 (55.71)	139 (59.66)	128 (54.24)
Top 100 domestic suppliers	13 (5.68)	18 (12.86)	8 (3.43)	26 (11.02)
Suppliers with joint venture	47 (20.52)	26 (18.57)	54 (23.18)	53 (22.46)
Foreign-owned suppliers	28 (12.23)	18 (12.86)	32 (13.73)	29 (12.29)
**Number (%) of products with:**				
Original brand	43 (18.78)	26 (18.57)	43 (18.45)	40 (16.95)
Generic	186 (81.22)	114 (81.43)	190 (81.55)	196 (83.05)
**Number (%) of products administered through:**				
Oral	141 (61.57)	89 (63.57)	151 (64.81)	162 (68.64)
Injectable	88 (38.43)	51 (36.43)	82 (35.19)	74 (31.36)

Note: *INN* - International Nonproprietary Name; *SD—*standard deviation.

### Changes in Unit Price of Medicines

**FIGURE 1:**
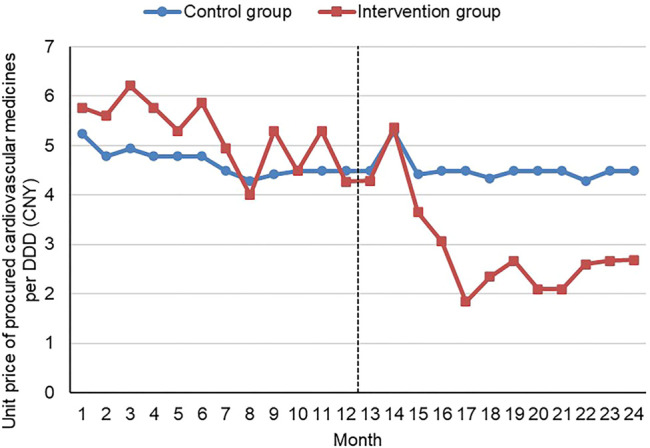
Median unit price per DDD of procured cardiovascular medicines by month Note: CNY—Chinese Yuan; DDD—defined daily dosage

The median price (DDD adjusted) of procured cardiovascular medicines decreased by 41.51% in the intervention group (Xiangyang) after the initiative, down from 5.30 Yuan in 2017 to 3.10 Yuan in 2018, compared with a 0.22% decrease (from 4.49 Yuan to 4.48 Yuan) in the control group (Yichang). The median unit price hovered at a high level prior to the introduction of the volume-price contract system in the intervention group. The new initiative resulted in a dramatic drop in the unit price from the third month to the fifth month after the introduction of the volume-price contract system, followed by a levelling off of around two and three Yuan. Over the study period, no significant changes in the median unit price were observed in the control group. The median unit price in the control group was lower prior to the new initiative but higher post the new initiative in comparison with the intervention group (Figure 1).

### Factors Associated With Changes in the Unit Price of Procured Cardiovascular Medicines

Considering the log link applied in the modelling, we made some transformations of the coefficients (Zhang et al., 2017).

The results of the difference-in-differences regression analyses showed that the volume-price contract initiative was associated with a 36.24% reduction (*p* = 0.006) in the unit price (DDD adjusted) of the procured cardiovascular medicines after adjustment for variations in other variables. In addition, therapeutic competition was associated with a 31.61% reduction (*p* = 0.002) in the unit price. No significant effects of generic competition were found (Table 4).

**TABLE 4:** Generalized linear regression model on unit price of all procured cardiovascular medicines with difference-in-differences analyses.

Variables	β coefficient	Robust product clustered standard error	z	*p*>|z|	95% confidence interval
Intervention effect (difference-in-differences)	**−0.45**	0.16	−2.73	**0.006**	−0.77 to -0.13
Generic competition (X1)	0.17	0.20	0.87	0.385	−0.22 to 0.57
(X1)^2^	−0.02	0.02	−0.63	0.526	−0.06 to 0.03
Therapeutic competition (X2)	**−0.38**	0.13	−3.03	**0.002**	−0.63 to -0.13
(X2)^2^	0.01	0.01	1.35	0.176	−0.00 to 0.02
Goodness-of-fit tests					
	Akaike Information Criterion (AIC) = 10.67				
	Bayesian Information Criterion (BIC) = -79,081.63				

Note: Bold values indicate regression coefficients with statistical significance (*p* < 0.05).

### Subgroup Analyses on Intervention Effects

The subgroup difference-in-differences analyses revealed that the intervention effects on the unit price of procured cardiovascular medicines were statistically significant for those with an original brand and those supplied by the top 100 domestic suppliers. The unit price from the top 100 domestic suppliers dropped by 82.80% (*p* = 0.001), while the unit price of those with an original brand dropped by 30.23% (*p* = 0.008) as a result of the volume-price contract arrangements. No significant intervention effects were observed for the generic medicines and other suppliers. No significant intervention effects were observed in subgroups of medicines categorized by administration routes (Table 5).

**TABLE 5:** Subgroup difference-in-differences analyses (generalized linear regression) on unit price of procured cardiovascular medicines.

Subgroup	N	Intervention effect					Goodness-of-fit tests	
		Coefficient	SE	Z	*p*>|z|	95%CI	AIC	BIC
**Suppliers**								
Domestic suppliers outside of top 100	7,278	0.21	0.23	0.91	0.363	−0.25 to 0.67	10.83	−32,420.92
Top 100 domestic suppliers	1,157	−**1.76**	0.52	−3.42	**0.001**	−2.78 to -0.75	8.39	−5,462.00
Suppliers with joint venture	3,561	−0.51	0.60	−0.85	0.393	−1.69 to 0.66	10.99	−10,200.43
Foreign-owned suppliers	3,925	−0.08	0.06	−1.25	0.210	−0.20 to 0.04	6.84	−31,932.43
**Medicines**								
Original brand	5,488	−**0.36**	0.13	-2.63	**0.008**	−0.62 to -0.09	6.59	-45,988.30
Generic	10,433	−0.28	0.18	−1.51	0.130	−0.64 to 0.08	11.32	-45,641.75
**Administration route**								
Injectable	4,366	0.17	0.19	0.87	0.382	−0.21 to 0.54	14.29	−23,176.32
Oral	11,555	−0.08	0.06	−1.40	0.162	−0.19 to 0.03	4.63	−100,023.10

Note: Bold values indicate regression coefficients with statistical significance (*p* < 0.05). *AIC -* Akaike information criterion; *BIC -* Bayesian information criterion; *CI -* confidence interval; *SE -* product clustered standard error.

## Discussion

Our study examined the impact of the volume-price contract initiative on the unit price of procured cardiovascular medicines through a natural experimental design involving 15,921 procurement records for 35 hospitals over a 2-year period. The generalized linear regression model with a difference-in-differences approach revealed that the volume-price contract arrangements contributed to a 36.24% drop in the unit price of procured cardiovascular medicines and a 31.61% drop in the unit price resulting from therapeutic competition after adjustment for variations in other variables. The medicines with an original brand and those supplied by the top 100 domestic suppliers were particularly sensitive to the new initiative, with a 30.23% and 82.80% drop in unit pricing, respectively.

The results indicate that the volume-price contract initiative offers an additional tool to reduce the unit price of medicines on top of the competition mechanism. Collective tendering and purchasing has been a common practice in most countries worldwide to source affordable medicines. The rationale lies in the theory of economies of scale (Li and Bai, 2019). With a large volume, the marginal cost for increasing production drops, which can result in a lowered average unit cost. Furthermore, a promised purchase volume brings certainty, which can help suppliers avoid or reduce some administrative and transaction costs. In the past, the awarded tenderers had to conduct market research, negotiate with individual health institutions, and promote their products in competition with other suppliers to win a purchase order. These costs, in particular the marketing costs, could be very high and had to be factored into consideration in the price setting (Ge, 2020; Huang and Tao, 2020). The new procurement arrangement now offered the awarded tenderers assurance of a large pooled purchase volume, giving them costing advantages in manufacturing and distributing the contracted products. This may even generate a flow-over effect on the surrounding regions through intensified price competition (Li and Bai, 2019), although we did not observe such a phenomenon in our study.

It is important to note that the impact of the volume-price contract initiative varies by supplier. The foreign-owned and joint ventures and the smaller domestic suppliers in this study were found to be less responsive to the new initiative in price setting than the top 100 domestic suppliers. The underlying reasons are not very clear. For small suppliers, their production capacity is limited, which may prevent them from participating in the large volume-based tendering. Unlikely their large counterparts, small suppliers do not have the advantage of economies of scale and may have limited space to cut costs. In addition, small domestic suppliers are most likely to be local. There may be a lack of incentives for them to reduce price under the protectionism of local governments (Wu et al., 2014).

Another interesting finding of this study is that generic medicines are less responsive in price setting to the volume-price contract system than those with an original brand. Generic medicines are always priced lower than their original-brand counterparts in the pharmaceutical retail market. The price gap between generic medicines and original brands, including cardiovascular medicines, is quite big in China (Zeng, 2013), which gives the original brands more room for price reduction. Indeed, most generic medicines are produced by small manufacturers in China. They tend to enter the retail market with low prices. The availability of lower-priced competitors can drive down the price of the original brands (Chapman et al., 2019). However, the original brands do not always engage in price competition with the generic medicines in China. They have occupied a large market share and are able to maintain higher prices due to longstanding concerns from the public about the quality of generic medicines. The perceived difference in the quality of medicines has weakened the competition effect between generic medicines and original brands (Chen and Rao, 2019).

The findings of this study have several policy implications. First, the effect of the volume-price contract initiative is effective in bringing down price only when the procurement volume is large enough. This imposes a serious challenge to the procurement of generic medicines as there are large numbers of suppliers but each occupies a small market share. The municipality-wide procurement volume may not be big enough to incentivize suppliers to cut the price of already lower-priced generic medicines. A higher (provincial or even national) level of pooled procurement arrangement can increase the procurement volume and create a competitive market. This may also encourage large manufacturers to produce generic medicines. In recent years, the national government in China has encouraged 11 provinces/regions to organize volume-based procurement for some generic medicines (Tang et al., 2019). Second, the medicines with a brand name are very responsive in price setting to the volume-price contract system, which can bring benefits in driving the quality improvement of generic medicines as their price gaps are shrinking. In 2016, the State Council of China released policy guidelines for establishing efficacy equivalence of generic medicines with an aim to resume consumer confidence in generic medicines through strengthened quality assurance mechanisms (The State Council of the People’s Republic of China, 2016).

To our knowledge, this is the first study of its kind in China to examine the impact of the volume-price contract system on the unit price of procured cardiovascular medicines. It provides additional evidence to the existing literature that advocates for collective tendering and purchasing of medicines based on volume and price. Data used in this study were extracted from the tendering platform, which had a large sample size and avoided sampling bias.

There are some limitations in this study. First, we could not exclude the potential impact of heterogeneity of medicines although the study was limited to cardiovascular medicines. The quality and efficacy information of the procured medicines was absent, preventing us from assessing the impacts of the new procurement system comprehensively apart from the unit price. The potential impact of the new supply arrangement on clinical services and patient care outcomes is unknown. However, this may not be an issue since the quality gap between generic medicines and originator brands is being gradually narrowed as seen in countries including China (Davit et al., 2009; Corrao et al., 2014; Jackevicius et al., 2016; The State Council of the People’s Republic of China, 2016). In addition, there is little difference in effectiveness or safety of different medicines for cardiovascular diseases (Wei et al., 2020). Future studies should take a patient perspective and cover a wider range of medicines as the new supply arrangement may have differing effects on the supply of different medicines. Second, each individual transaction was treated as a unit of analysis without consideration of the duration of contract (because of the lack of variations) and how previous contracts informed subsequent procurement from the same supplier (because of data unavailability). Third, although China’s pharmaceutical supply system has been improved substantially by the strong regulations from the government (Yan et al., 2018), there is still problem of fragmentation in the regulatory, which exacerbates the lack of transparency in the pharmaceutical system (Hu and Mossialos, 2016). In addition, China’s pharmaceutical market has been characterized by dispersion and low concentration, which leads to uneven pricing problems of pharmaceuticals (Hu and Mossialos, 2016). Thus, the generalization of the conclusions to other settings should be conducted with caution.

## Conclusion

In conclusion, the volume-price contract initiative is effective in reducing the unit price of procured cardiovascular medicines. The effect remained significant after adjustment for the competition effects. However, the impacts of the new initiative vary by medicine and supplier. The cardiovascular medicines with an original brand and the top 100 domestic suppliers were more responsive to the new initiative than others. Increasing procurement volumes may further enhance the impact of the volume-price contract system. But local protectionism can create a great barrier for cross-region collaborations.

## Data Availability

The raw data supporting the conclusion of this article will be made available by the authors, without undue reservation.
