# Supplementary material for: Effects of Volume-Price Contracts on Pharmaceutical Prices: A Retrospective Comparative Study of Public Hospitals in Hubei of China
Source: Front Pharmacol. 2021 Oct 14;12:741671. doi: 10.3389/fphar.2021.741671 (PMC8552023; doi:10.3389/fphar.2021.741671)
Supplement: Supplementary file 1 [file Table1.DOCX]

Supplementary Material

**Table 1 Results of (unweighted) TOPSIS ranking**

|  | GDP (100 million CNY) | Per capita GDP (CNY) | Population size (10,000) | Per capita disposable income (CNY) | Number of health institutions | Number of hospital beds (10,000) | Number of licensed (assistant) doctors (10,000) | Number of skilled health workers | TOPSIS score | Rankings |
| --- | --- | --- | --- | --- | --- | --- | --- | --- | --- | --- |
| Wuhan | 13410.340 | 123831 | 1089.29 | 38642 | 6071 | 9.16 | 3.630 | 127413 | 1 | 1 |
| Xiangyang | 4064.900 | 71990 | 565.40 | 24030 | 3731 | 3.65 | 1.390 | 39591 | 0.378043889 | 2 |
| Yichang | 3857.170 | 93331 | 413.56 | 24182 | 3013 | 2.82 | 1.090 | 38275 | 0.341987109 | 3 |
| Huanggang | 1921.830 | 30356 | 634.10 | 17658 | 4220 | 3.59 | 1.340 | 43947 | 0.338993532 | 4 |
| Jingzhou | 1922.180 | 33902 | 564.17 | 22261 | 3297 | 3.03 | 1.260 | 32358 | 0.289476477 | 5 |
| Shiyan | 1632.320 | 47820 | 341.80 | 18716 | 2819 | 2.56 | 0.950 | 27405 | 0.244275316 | 6 |
| Enshi | 801.230 | 23892 | 336.10 | 15259 | 3067 | 2.40 | 0.800 | 21872 | 0.216599658 | 7 |
| Jingmen | 1664.170 | 57357 | 290.15 | 24049 | 1992 | 1.69 | 0.730 | 21454 | 0.19831306 | 8 |
| Xiaogan | 1742.230 | 35486 | 491.50 | 22256 | 168 | 2.05 | 0.880 | 24187 | 0.176632672 | 9 |
| Huangshi | 1479.400 | 59943 | 247.05 | 24968 | 1317 | 1.57 | 0.610 | 14087 | 0.167371133 | 10 |
| Xianning | 1234.860 | 48798 | 253.51 | 20310 | 1303 | 1.44 | 0.670 | 17147 | 0.152125464 | 11 |
| Ezhou | 905.920 | 84452 | 107.69 | 24214 | 497 | 0.61 | 0.220 | 8336 | 0.147362831 | 12 |
| Qianjiang | 671.860 | 69731 | 101.06 | 22689 | 710 | 0.42 | 0.210 | 5748 | 0.117777714 | 13 |
| Suizhou | 935.720 | 42414 | 221.05 | 20069 | 1363 | 1.14 | 0.430 | 10197 | 0.117441689 | 14 |
| Xiantao | 718.660 | 62792 | 114.10 | 22963 | 991 | 0.53 | 0.260 | 6868 | 0.115781043 | 15 |
| Tianmen | 528.250 | 40039 | 128.35 | 20486 | 1043 | 0.61 | 0.270 | 6337 | 0.082872683 | 16 |
| Shennongjia | 255.108 | 33196 | 1.14 | 16482 | 84 | 0.05 | 0.021 | 466 | 0.022042567 | 17 |

Note: Data were extracted from the statistical reports of Hubei province and its municipalities. *CNY -* Chinese yuan; *GDP -* gross domestic product; *TOPSIS -* technique for order performance by similarity to ideal solution.
